# A Vaccination Crusade

**Published:** 1896-12-12

**Authors:** 


### The Hospital

A Journal of

The Medical Sciences and Hospital Administration, with

AN EXTRA NURSING SUPPLEMENT.

Vol. XXI.—No. 533.] [Dec. 12, 1896.

### A Vaccination Crusade.

After a lapse of a hundred years it is proposed to raise a national—why not an international?—memorial to Edward Jenner. That the civilised world should raise a monument to the memory of Jenner is to pay a debt long overdue, and a debt which has borne interest, the value of which can only be calculated in the terms of millions—millions, not of pounds sterling, but of human lives. If a memorial is to be raised to Jenner after the world has deliberated on his claims for so long a period as a hundred years it must fulfil, at least, two conditions—it must be public, and not merely professional; and it must be adequate and worthy, not poor and mean.

The form which a given monument, when decided upon, shall assume, is often a very difficult point to decide. It is hardly so in the present case, if national, or international, gratitude will make an effort and rise to a great occasion. Vaccination has been proved, by more critical processes than any scientific or religious doctrines have ever been submitted to before, to be disease-preventing and life-saving on the widest possible scale; and that being so, the natural monument which an enlightened civilisation would be best pleased with would be the establishment of some organised propaganda, which should have for its object the conferring of the benefits of vaccination upon all mankind.

The medical profession display an amount of disinterested feeling and energy on behalf of vaccination which is really surprising; and which is in flat contradiction to some of the most universally secured of the philosophic theories of the motives of human action. The average man, in his daily life, follows perseveringly the lines of his own interests; and does so, not only without condemnation, but with universal approval. The medical man, when he encourages vaccination, *ipso facto* does his best to "kill the goose which lays the golden eggs." Let there be no mistake at all about this point. The priests of Diana defended her shrine because, as they said to one another, "by this craft we have our wealth." The modern doctor, in promoting vaccination, is entitled to say that the more successful his efforts the greater will be his own losses as a practising physician.

The non-professional world can hardly be expected to set about establishing a vaccination propaganda on its own account, because it has neither the requisite knowledge nor the animating spirit for such a work. But what it can do, and must do as a matter of course, is to supply the "sinews of war." The members of the medical profession will give all they can give—time, knowledge, skill, enthusiasm; but that they should, in addition, take money out of their pockets which they know will be used to scare away from these pockets still more money, is a course which no fair-minded man can possibly expect them to pursue. If Jenner had conferred an advantage upon doctors; if he had discovered a means of curing small-pox instead of preventing it; and so of bringing work and remuneration to the profession, it would have been quite consistent that doctors should erect his monument and pay for the preservation of his fame. But it is the public, and the public exclusively, which profits by Jenner's great discovery, and the public, if it can be made to understand its obligations, will certainly not be niggardly in its gratitude and rewards.

The Jenner Society, as is right, is moving in this matter, and its hon. secretary, Dr. Francis Bond, may be trusted to do whatever is possible with the limited resources at his disposal. But the Jenner Society is to a large extent a local society, and its small funds are obtained for the most part within the county of Gloucester alone. It can hardly be expected that a world-wide movement shall have its centre at Gloucester. But if the principal centre must be at Gloucester, then other centres, in London, Edinburgh, Dublin, New York, Paris, Vienna, Berlin, St. Petersburg, and elsewhere, should be established without delay. Unfortunately Dr. Bond does not outline a scheme of procedure. But it will be necessary that such a scheme be prepared, and that enthusiastic Jennerians be enrolled among medical men throughout the length and breadth of the civilised world.

Fortunately for the success of the Jenner memorial, there is a determined opposition. An opposed cause is almost always a successful cause; and nothing has tended so much to awaken the interest of the medical profession as the ignorant and fanciful attacks which a few hot-headed persons, followed by crowds more ignorant and fanciful than themselves, have made upon vaccination. The Royal Commission has taken all the wind out of the sails of these gentlemen; and if the medical profession, following the lead of the Jenner Society, will now take up the matter with enthusiasm, there can hardly be any doubt that the great European and American public will respond with enthusiasm, and that the cause of vaccination will receive an impetus such as it has never experienced before.

